# Prospective Study of Preferred Versus Actual Place of Death Among Swedish Palliative Cancer Patients

**DOI:** 10.1177/10499091231213640

**Published:** 2023-11-06

**Authors:** Jonas Nilsson, Stefan Bergström, Hampus Hållberg, Anders Berglund, Michael Bergqvist, Georg Holgersson

**Affiliations:** 1 Center for Research & Development, Uppsala University/ County Council of Gävleborg, Gävle Hospital, Gävle, Sweden; 2 Department of Radiation Sciences & Oncology, Umeå University Hospital, Umeå, Sweden; 3 Department of Radiology, Gävle Hospital, Gävle, Sweden; 4 EpiStat, Uppsala, Sweden; 5 Department of Immunology Genetics and Pathology, Section of clinical and experimental oncology, Uppsala University Hospital, Uppsala, Sweden

**Keywords:** palliative care, cancer patients, place of death, home death, questionnaire, prospective study

## Abstract

**Background:** The place of death of cancer patients is an important aspect of end-of-life care. However, little research has been conducted regarding factors that may influence the preferred and actual place of death in cancer patients and whether the patients die at their preferred place of death. In this study, we aimed to investigate the preferred and actual place of death for palliative cancer patients, and factors influencing these variables. **Methods:** Patients diagnosed with cancer and admitted to a palliative care team across three Swedish cities between 2019 and 2022 were asked for participation. Participants completed a questionnaire capturing sociodemographic data and preferred place of death. Further data regarding age, sex, and cancer type were collated at inclusion, and the actual place of death recorded for those deceased by 5-May-2023. **Results:** The study included 242 patients. A majority (79%) wanted to die at home which was the actual death location for 76% of the patients. When the place-of-death decision was made by the patient alone, 75% chose home, compared to 96% when decided jointly with relatives—a statistically significant variation (p = 0.0037). For the patients who wanted to die at home, 80% actually died at home, with insignificant disparities among subgroups. **Conclusions:** Most palliative cancer patients in this Swedish cohort preferred and achieved death at home. Involving relatives in decision-making may influence the preferred place of death, however larger studies are needed to comprehensively assess factors affecting the preferred and actual place of death in different subgroups of patients.

## Introduction

The place of death is an important quality aspect of end-of-life care.^1^ The needs of terminally ill patients differ, and therefore the preferred place of death of patients and the reasons behind these preferences may vary. Previous studies, as well as a systematic review of the subject,^2^ have shown that most cancer patients prefer to die at home rather than at a hospital to spend their last time in a familiar environment.^3-5^ There are also indications that dying at home is associated with a better death for most patients.^6^ In 1 study comparing places of death in different countries, there was a median of 54% of hospital deaths and 18% of deaths in residential aged care facilities.^7^ Also, the proportion of hospital deaths varied considerably between different countries which may be due to cultural reasons as well as differences in health care systems in different countries.^7,8^ Death certificate data from all deaths in 2002 in Sweden showed that 85% of all cancer deaths occurred in a hospital.^9^ In a more recent population-based study based on death certificate data from all deceased patients in Sweden in 2012, 42% of all deaths occurred in hospitals, 18% occurred at home and 38% in nursing home facilities, suggesting a trend over time in Sweden of decreasing likelihood of hospital death.^10^ However, the opposite trend has also been observed in other countries.^11^ There are patients who prefer to die at a hospital or a nursing home because of hope of receiving treatment or to reduce the burden of care of their family.^12^ Also, limitations regarding level of care and complex symptom control makes end-of-life care at home unsuitable for some patients. Therefore, it may be inappropriate to assume that all end-of-life care should be organized in a home setting and the preferences and needs of the patient should always be considered. However little research has been conducted regarding preferred vs actual place of death in cancer patients and the factors that influence these variables. In 1 retrospective study investigating patients admitted to advanced medical home care in Stockholm, Sweden, it was shown that of the patients who expressed a preferred place of death, 75% wanted to die at home and 80% had their wishes fulfilled.^13^ In this study, the wish to die at home was the only factor significantly influencing the place of death. Other studies, from different countries and time periods, have shown a congruence between preferred and actual place of death of 31%–73%,^14-21^ suggesting significant variations between different populations. In these studies, various other factors that the patient’s preferences have been significantly associated with the actual place of death. To improve the quality of end-of-life care, it is important that patients are able to die in their preferred locations and that health care workers help patients to achieve this. The aim of this prospective trial was to investigate the preferred and the actual place of death of palliative cancer patients and to assess the impact of various socio-demographic factors on these variables.

## Methods

### Setting

Palliative home-care teams in Sweden are organized in different ways in different parts of the country depending on regional preconditions. They have in common that they offer hospital-like care at home, including visits of nurses, palliative care physicians and paramedics with the aim to provide good palliative care to patients in their own home, to avoid hospital admissions and to make it possible for the patient to live at home for as long as possible during the end of life. Usually, only patients with symptoms that require specialized palliative care are enrolled in a palliative home-care team but the practice varies between different regions. Patients who need health care services or nursing care at home but who do not need specialized palliative care can receive help from municipal home care (Sw: hemsjukvård). Furthermore, municipal domestic service (Sw: hemtjänst), offering non-medical care, is accessible to all elderly and disabled in Sweden for a subsidized fee.

This study was conducted in the Swedish municipalities of Gävle, Bollnäs and Avesta. Together they have a population of 153,000 inhabitants which makes up about 1.5% of the total population in Sweden. Each of the municipalities has a central city with the same name as the municipality in which a majority of the population lives. However, there is also a sizable rural population in each municipality. Each of these 3 municipalities has its own palliative home care team which resides in the central city.

### Data collection

Patients aged 18 years or above diagnosed with cancer in a palliative setting who were enrolled in a palliative home-care team in the Swedish municipalities of Gävle, Bollnäs and Avesta between 2019 and 2022 were asked to participate in the study.

Exclusion criteria were cognitive impairment, or any co-morbidity or communication issue that obstructed the ability to fill in a questionnaire according to a medical assessment by the study staff.

Patients who gave their written consent to participate were given a questionnaire (Supplemental Table 1) which consisted of 5 multiple choice questions regarding preferred place of death and whether this decision was made by the patient alone or together with relatives, marital status, need of domestic service or health care at home and need of assistance from relatives. The questionnaire was completed either by the patient or by health care personnel together with the patient. Also, demographic data regarding age and sex and type of cancer were collected for all patients at the time of inclusion. The place of death was recorded for all the patients in the study who died before the last follow up date of 5-May-2023. Ethical approval was received on 8-Nov-2017 from The Swedish Ethical Review Authority (Dnr 2017/271).

### Statistical analysis

Patients’ characteristics were presented with descriptive statistics where categorical variables were presented as absolute numbers and percentages. In a next step, preferred place of death, and actual place of death was described by patient characteristics and statistically tested using the Chi-square test (and fisher’s exact test if the expected numbers were less than 5). In a subsequent step, the comparison of preferred and actual place of death was testing using the Chi-Square test (or Fishers exact test). All tests were 2-sided and statistical significance was considered with a *P*-value less than 5%. The statistical analyses were performed using R version 4.2.2. (R basis for statistical calculation, Vienna University of Economics and Business, Vienna, Austria).

## Results

In total, 663 patients were evaluated for participation in the study. Of these, 421 patients had to be excluded due to cognitive impairment (n = 303), comorbidity (n = 26), communication issues (n = 19), declined participation by the patient (n = 15) or other reasons (n = 58). Thus, 242 patients were included in the study, 225 from Gävle, 10 from Avesta and 7 from Bollnäs. Of these 114 (47%) were women. The median age was 76 years (range 35-94 years). About half of the patients (54%) were married or lived permanently together with a partner. The 5 most common cancer types were pancreatic cancer (19%), lower gastrointestinal cancer (15%), upper gastrointestinal cancer (13%), prostate cancer (10%) and lung cancer/mesothelioma (10%). Most of the patients had health care at home (93%) and a smaller portion of the patients had home care and domestic service (26%). In addition, most of the patients (75%) got assistance from their relatives. Most of the patients preferred to die at home (79%) and some patients (14%) did not know where they wanted to die. In most cases (78%) the decision regarding preferred place of death was made by the patient alone. Regarding actual place of death, most patients (76%) died at home. The descriptive data are summarized in Table 1.

**Table 1.** Patient Characteristics.

Characteristic	Number
Total	242 (100%)
Sex	
Female	114 (47.1%)
Male	128 (52.9%)
Age (years)	
<76 years	114 (47.1%)
≥76 years	128 (52.9%)
Diagnosis	
Brain tumor	4 (1.7%)
Breast cancer	15 (6.2%)
Lung cancer/mesothelioma	24 (9.9%)
Lower gastrointestinal cancer	35 (14,5%)
Upper gastrointenstinal cancer	31 (12.8%)
Pancreatic cancer	46 (19.0%)
Hepatocellular carcinoma	8 (3.3%)
Gynecological cancer	11 (4.5%)
Prostate cancer	24 (9.9%)
Urological cancer (excluding prostate)	12 (5.0%)
Skin cancer/malignant melanoma	7 (2.9%)
Lymphoma/myeloma	5 (2.1%)
Sarcoma	5 (2.1%)
Cancer with unkown primary	6 (2.5%)
Other cancer	9 (3.7%)
Marital status	
Married	103 (42.6%)
Partner, living together	26 (10.7%)
Partner in different household	11 (4.5%)
No partner	100 (41.3%)
Other	1 (.8%)
Home service	
None	16 (6.6%)
Home care	162 (66.9%)
Domestic service	1 (.4%)
Home care and domestic service	63 (26.0%)
Help from relatives	
No need of help	52 (21.8%)
Takes care of self but would like help from relatives	6 (2.5%)
Gets help from relatives in the same household	93 (38.9%)
Gets help from relatives that live in a different household	86 (36.0%)
Other	2 (.8%)
Choice of preferred place of death	
Patient’s own decision	189 (78.1%)
Decision made together with relatives	53 (21.9%)
Preferred place of death	
At home	190 (78.5%)
Hospital	6 (2.5%)
Nursing home	9 (3.7%)
Other	3 (1.2%)
Don’t know	34 (14.0%)
Actual place of death	
At home	184 (76.0%)
Hospital	27 (11.2%)
Nursing home	30 (12.4%)
Missing	1 (.4%)

For patients where the decision regarding preferred place of death was made by the patient alone, 74% wanted to die at home as compared with 96% when the decision was made together with relatives. This difference was statistically significant (*P* = .0037). However, there was no statistically significant difference when comparing actual place of death between these 2 groups. There was no statistically significant difference regarding preferred or actual place of death with respect to sex, age, marital status, type of tumor or health care at home. Data regarding preferred and actual place of death in different subgroups are presented in Tables 2 and 3.

**Table 2.** Preferred Place of Death.

Characteristic	At Home	Hosptial	Nursing Home	Other	Don’t Know	*P*-value
Sex						.32
Female	89 (78.1%)	4 (3.5%)	3 (2.6%)	3 (2.6%)	15 (13.2%)	
Male	101 (78.9%)	2 (1.6%)	6 (4.7%)	0 (0%)	19 (14.8%)	
Age (years)						.48
<76 years	94 (82.5%)	3 (2.6%)	3 (2.6%)	2 (1.8%)	12 (10.5%)	
≥76 years	96 (75.0%)	3 (2.3%)	6 (4.7%)	1 (.8%)	22 (17.2%)	
Diagnosis						
Brain tumor	4 (100%)	0 (0%)	0 (0%)	0 (0%)	0 (0%)	
Breast cancer	12 (80.0%)	0 (0%)	0 (0%)	1 (6.7%)	2 (13.3%)	
Lung cancer/mesothelioma	18 (75.0%)	1 (4.2%)	1 (4.2%)	0 (0%)	4 (16.7%)	
Lower gastrointestinal cancer	28 (80.0%)	0 (0%)	0 (0%)	0 (0%)	7 (20.0%)	
Upper gastrointenstinal cancer	24 (77.4%)	1 (3.2%)	1 (3.2%)	0 (0%)	5 (16.1%)	
Pancreatic cancer	33 (71.7%)	2 (4.3%)	2 (4.3%)	2 (4.3%)	7 (15.2%)	N/A
Hepatocellular carcinoma	6 (75.0%)	0 (0%)	0 (0%)	0 (0%)	2 (25.0%)	
Gynecological cancer	9 (81.8%)	0 (0%)	0 (0%)	0 (0%)	2 (18.2%)	
Prostate cancer	19 (79.2%)	1 (4.2%)	3 (12.5%)	0 (0%)	1 (4.2%)	
Urological cancer (excluding prostate)	10 (83.3%)	0 (0%)	0 (0%)	0 (0%)	2 (16.7%)	
Skin cancer/malignant melanoma	5 (71.4%)	0 (0%)	1 (14.3%)	0 (0%)	1 (14.3%)	
Lymphoma/myeloma	3 (60.0%)	0 (0%)	1 (20.0%)	0 (0%)	1 (20.0%)	
Sarcoma	5 (100%)	0 (0%)	0 (0%)	0 (0%)	0 (0%)	
Cancer with unkown primary	6 (100%)	0 (0%)	0 (0%)	0 (0%)	0 (0%)	
Other cancer	8 (88.9%)	1 (11.1%)	0 (0%)	0 (0%)	0 (0%)	
Marital status						.42
Married	82 (79.6%)	1 (1.0%)	3 (2.9%)	2 (1.9%)	15 (14.6%)	
Partner, living together	17 (65.4%)	2 (7.6%)	3 (11.5%)	0 (0%)	4 (15.4%)	
Partner in different household	11 (100%)	0 (0%)	0 (0%)	0 (0%)	0 (0%)	
No partner	78 (78.0%)	3 (3.0%)	3 (3.0%)	1 (1.0%)	15 (15.0%)	
Home service						.11
None	10 (62.5%)	0 (0%)	1 (6.3%)	0 (0%)	5 (31.3%)	
Home care	132 (81.5%)	5 (3.1%)	4 (2.5%)	1 (.6%)	20 (12.4%)	
Domestic service	0 (0%)	0 (0%)	0 (0%)	0 (0%)	1 (100%)	
Home care and domestic service	48 (76.2%)	1 (1.6%)	4 (6.3%)	2 (3.2%)	8 (12.7%)	
Help from relatives						.64
No need of help	39 (75.0%)	0 (0%)	3 (5.8%)	1 (1.9%)	9 (17.3%)	
Takes care of self but would like help from relatives	4 (66.7%)	0 (0%)	0 (0%)	0 (0%)	2 (33.3%)	
Gets help from relatives in the same household	77 (82.8%)	4 (4.3%)	2 (2.2%)	0 (0%)	10 (10.8%)	
Gets help from relatives that live in a different household	67 (77.9%)	2 (2.3%)	3 (3.5%)	2 (2.3%)	12 (14.0%)	
Other	2 (100%)	0 (0%)	0 (0%)	0 (0%)	0 (0%)	
Choice of preferred place of death						.0037
Patient’s own decision	139 (73.5%)	5 (2.7%)	9 (4.8%)	3 (1.6%)	33 (17.5%)	
Decision made together with relatives	51 (96.2%)	1 (1.9%)	0 (0%)	0 (0%)	1 (1.9%)

**Table 3.** Actual Place of Death.

Characteristic	At Home	Hosptial	Nursing Home	Missing	*P*-value
Sex					
Female	87 (76.3%)	14 (12.3%)	13 (11.4%)	0 (0%)	.89
Male	97 (75.8%)	13 (10.2%)	17 (13.3%)	1 (.8%)	
Age (years)					
<76 years	86 (75.4%)	15 (13.6%)	12 (10.5%)	1 (.9%)	.69
≥76 years	98 (76.6%)	12 (9.4%)	18 (14.1%)	0 (0%)	
Diagnosis					
Brain tumor	2 (50.0%)	0 (0%)	2 (50.0%)	0 (0%)	
Breast cancer	12 (80.00%)	2 (13.3%)	1 (6.7%)	0 (0%)	
Lung cancer/mesothelioma	19 (79.2%)	1 (4.2%)	4 (16.7%)	0 (0%)	
Lower gastrointestinal cancer	27 (77.1%)	2 (5.7%)	6 (17.1%)	0 (0%)	
Upper gastrointenstinal cancer	21 (67.4%)	7 (22.2%)	3 (11.1%)	0 (0%)	
Pancreatic cancer	34 (73.9%)	7 (15.2%)	5 (10.9%)	0 (0%)	N/A
Hepatocellular carcinoma	7 (87.5%)	1 (12.5%)	0 (0%)	0 (0%)	
Gynecological cancer	8 (72.7%)	2 (18.2%)	1 (9.1%)	0 (0%)	
Prostate cancer	18 (75.0%)	2 (8.3%)	4 (16.7%)	0 (0%)	
Urological cancer (excluding prostate)	11 (91.7%)	0 (0%)	1 (8.3%)	0 (0%)	
Skin cancer/malignant melanoma	5 (71.4%)	0 (0%)	2 (28.6%)	0 (0%)	
Lymphoma/myeloma	4 (80.0%)	0 (0%)	1 (20.0%)	0 (0%)	
Sarcoma	5 (100%)	0 (0%)	0 (0%)	0 (0%)	
Cancer with unkown primary	5 (83.3%)	1 (16.7%)	0 (0%)	0 (0%)	
Other cancer	6 (66.7%)	2 (22.2%)	0 (0%)	1 (11.1%)	
Marital status					.41
Married	83 (80.6%)	9 (8.7%)	11 (10.7%)	0 (0%)	
Partner, living together	19 (73.1%)	5 (19.2%)	2 (7.7%)	0 (0%)	
Partner in different household	8 (72.7%)	0 (0%)	3 (27.3%)	0 (0%)	
No partner	72 (72.0%)	13 (13.0%)	14 (14.0%)	1 (1.0%)	
Home service					.75
None	14 (87.5%)	1 (6.3%)	1 (6.3%)	0 (0%)	
Home care	122 (75.3%)	20 (12.4%)	20 (12.4%)	0 (0%)	
Domestic service	1 (100%)	0 (0%)	0 (0%)	0 (0%)	
Home care and domestic service	47 (74.6%)	6 (9.5%)	9 (14.3%)	1 (1.6%)	
Help from relatives					.003
No need of help	41 (78.9%)	1 (1.9%)	9 (17.3%)	1 (1.9%)	
Takes care of self but would like help from relatives	3 (50.0%)	2 (33.3%)	1 (16.7%)	0 (0%)	
Gets help from relatives in the same household	77 (82.8%)	12 (12.9%)	4 (4.3%)	0 (0%)	
Gets help from relatives that live in a different household	61 (70.9%)	10 (11.6%)	15 (17.4%)	0 (0%)	
Other	1 (50.0%)	1 (50.0%)	0 (0%)	0 (0%)	
Choice of preferred place of death					.78
Patient’s own decision	141 (74.6%)	22 (11.6%)	25 (13.2%)	1 (.5%)	
Decision made together with relatives	43 (81.1%)	5 (9.4%)	5 (9.4%)	0 (0%)	

**Figure 1.**
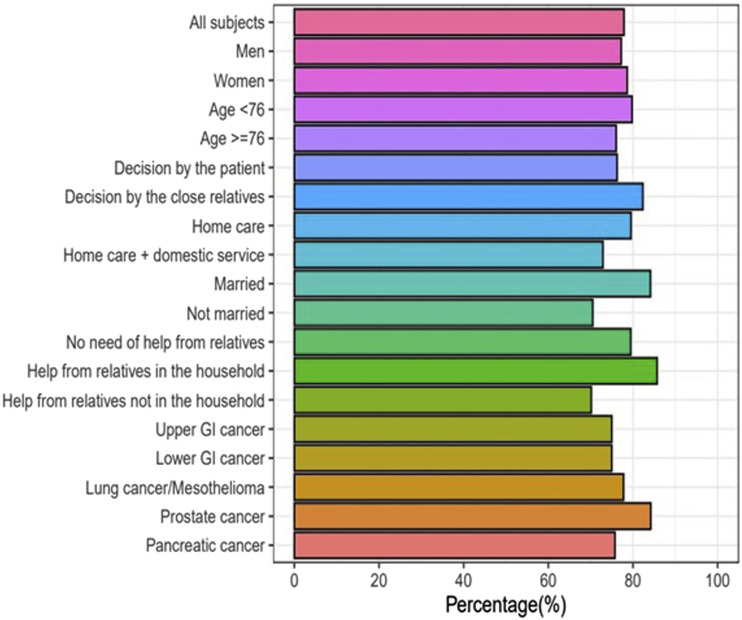
Percentage of home deaths of patients wanting to die at home in different subgroups.

When comparing preferred and actual place of death it was found that of the 190 patients who wanted to die at home, 148 (80%) died at home. Of the 9 patients who wanted to die at a nursing home, 4 (44%) died at a nursing home whereas 5 (56%) died at home. Of the 6 patients who wanted to die at a hospital, 4 (67%) died at a hospital whereas 1 (17%) died at home and 1 (17%) died at a nursing home. Of the 34 patients who did not know where they wanted to die, 29 (85%) died at home, 4 (12%) died at a hospital and 1 (3%) died at a nursing home. A comparison of preferred and actual place of death is presented in Table 4. The proportion of patients whose preferred and actual death place was at home was similar across different subgroups with respect to sex, age, marital status, or health care at home. A comparison of percentages of home deaths of patients wanting to die at home in different subgroups is presented in Figure 1. There were no statistically significant differences in the prevalence of home deaths in these subgroups.

**Table 4.** Comparison of Preferred and Actual Place of Death.

	Preferred Place of Death						
	At Home	Hospital	Nursing Home	Other	Don’t Know	Total	*P*-value
Actual place of death							.0006
At home	148 (80.4%)	1 (.54%)	5 (2.7%)	1 (.54%)	29 (15.8%)	184 (76.0%)	
Hospital	18 (66.7%)	4 (14.8%)	0 (0%)	1 (3.7%)	4 (14.8%)	27 (11.2%)	
Nursing home	23 (76.7%)	1 (3.3%)	4 (13.3%)	1 (3.3%)	1 (3.3%)	30 (12.4%)	
Missing	1 (100.00%)	0 (0%)	0 (0%)	0 (0%)	0 (0%)	1 (.41%)	
Total	190 (78.5%)	6 (2.5%)	9 (3.7%)	3 (1.2%)	34 (14.1%)	242 (100%)

## Discussion

In this prospective study of palliative cancer patients enrolled in a palliative home-care team setting in the Swedish municipalities of Gävle, Bollnäs and Avesta, we have shown that most of the patients (79%) wanted to die at home. Furthermore, most patients actually died at home (76%) whereas the percentage of patients who died at a hospital or a nursing home was much lower (23%).

Of the patients who wanted to die at home, 80% actually died at home. Very few patients in our study wanted to die at a hospital or a nursing home and in the end only about 1 10th of the patients actually died at a hospital or a nursing home. However, due to the low number of patients in these groups it is hard to draw any certain conclusions from these data. A minority of the patients (14%) did not know where they wanted to die and of these patients most (85%) died at home.

The proportion of patients who wanted to die at home was similar across different subgroups, however when the decision was made together with relatives, the proportion of patients who wanted to die at home was significantly higher (96%) as compared to when the decision was made by the patient alone (75%). The reason for this is unclear but could be due to that the patients who make their decision regarding place of death together with relatives have a higher confidence that they will get the support necessary to be able to spend their last days at home. However, when looking at the actual place of death, there was no significant difference in the frequency of home death between patients who made their decision together with relatives as compared to when the decision was made by the patient alone. Neither did the other investigated factors (sex, age, marital status, type of tumor, assistance from relatives or health care at home) affect the likelihood of home death. Of note in our study was that all brain tumor patients wished to die at home, however the wish was only realized in 2 out of 4 patients. However, the small number of patients makes this finding hard to interpret. Results from previous studies suggest that several factors influence the place of death in cancer patients. In a systematic review from 2006 including 58 studies with over 1.5 million cancer patients from 13 different countries it was found that 6 factors were strongly associated with home death; low functional status, patient preferences, home care, the intensity of home care, living with relatives and extended family support.^22^ Other factors associated with place of death has been proposed in recently published studies including marital status, the primary caregiver’s relationship with the patient and pain score.^19,20^

This study provides unique insights as it is to our knowledge the first study of Swedish cancer patients that has examined the preferences and actual death place of patients in a prospective manner. The percentage of patients who died at home is, by international comparison, high and an indicator of high quality of end-of-life care. In a multi-national comparison of places of death including 21 countries, there was a median of 54% of hospital deaths and 18% of deaths in residential aged care facilities.^7^ Also, there were large variations between different countries with 78% hospital deaths in a Japanese population as compared with 11% in an Albanian population. In another multi-national study, the percentage of cancer deaths occurring at home in several Western European countries during the years 2002-2003 ranged from 12.8% in Norway to 45.4% in the Netherlands.^8^ Generally, higher proportions of home deaths can be seen in more recently published studies; however the numbers vary cosiderably.^13,18-20^

Regarding the congruence between preferred and actual death place, previous studies have shown that between 31%–80% of patients achieved their preference for place of death.^13-21^ In previous studies using a prospective interview or questionnaire-based method similar to the present study, the congruence between preferred and actual death place was 48%–72%.^16-18,21^ The high level of congruence (80%) between preferred and actual death place in the present study is encouraging and similar to what was reported from a retrospective study of patients admitted to an advanced medical home care unit in Stockholm, Sweden.^13^ The differences observed between this study and previous studies may be due to cultural reasons^12^ as well as due to differences in access to palliative care specialists, nursing support, and equipment such as hospital beds.^23^

A considerable strength of the present study is the relatively large number of palliative cancer patients that have been included in a prospective manner. However, there are also limitations with the study that needs mentioning. One limitation is the relatively limited geographical area that has been subjected to the study and the results may be different in other areas of the country. Also, almost 2 thirds of the patients who were evaluated for the study had to be excluded, mainly due to cognitive impairment, which could possibly lead to selection bias. Another limitation of the study is the relatively few patients who wanted to die at other places than at home which makes it hard to draw conclusions regarding factors that influence the decisions of these patients. Another noteworthy aspect of the present study is that it was in large parts conducted during the COVID-19 pandemic. In theory, this may have affected the participants’ preferred place of death since being at home could be perceived as safer due to less risk of contagion, compared to being at a hospital or a nursing home.

## Conclusion

In conclusion, the findings of this study show that 79% of the Swedish palliative cancer patients included in this study wanted to die at home and 80% of these patients actually died at home, which are encouraging findings. We also found that patients who make their decision together with relatives are more likely to want to die at home whereas the actual place of death does not differ between these groups. Other factors such as sex, age, marital status, type of tumor, assistance from relatives or health care at home does not seem to impact the preference or likelihood of home death. These findings give valuable insights into the area of end-of-life care and the place of death preferences in palliative cancer patients in a Swedish context.

## Supplemental Material

Supplemental Material - Prospective Study of Preferred Versus Actual Place of Death Among Swedish Palliative Cancer Patients Supplemental Material for Prospective Study of Preferred Versus Actual Place of Death Among Swedish Palliative Cancer Patients by Jonas Nilsson, Stefan Bergström, Hampus Hållberg, Anders Berglund, Michael Bergqvist, and Georg Holgersson in American Journal of Hospice and Palliative Medicine

## Data Availability

Data relating to this research project can be obtained by contacting the corresponding author at georg.holgersson@akademiska.se

## References

[1] Clayton JM Butow PN Arnold RM Tattersall MH . Discussing life expectancy with terminally ill cancer patients and their carers: a qualitative study. Support Care Cancer : Official Journal of the Multinational Association of Supportive Care in Cancer. 2005;13(9):733-742. doi:10.1007/s00520-005-0789-4 15761699

[2] Nilsson J Blomberg C Holgersson G Carlsson T Bergqvist M Bergstrom S . End-of-life care: Where do cancer patients want to die? A systematic review. Asia Pac J Clin Oncol. 2017;13(6):356-364. doi:10.1111/ajco.12678 28294576

[3] Higginson IJ Sen-Gupta GJ . Place of care in advanced cancer: a qualitative systematic literature review of patient preferences. Journal of Palliative Medicine. 2000;3(3):287-300. doi:10.1089/jpm.2000.3.287 15859670

[4] Westfall K Moore D Meeneghan M Jarr S Valgus J Bernard S . The Impact on Resource utilization of supportive care consults on patients at the university of North Carolina hospital, 2010-2012. Journal of Palliative Medicine. 2018;21(2):216-219. doi:10.1089/jpm.2016.0482 28813627

[5] Decker SL Higginson IJ . A tale of two cities: factors affecting place of cancer death in London and New York. Eur J Publ Health. 2007;17(3):285-290. doi:10.1093/eurpub/ckl243 17068001

[6] Barbera L Paszat L Chartier C . Death in hospital for cancer patients: An indicator of quality of end-of-life care. Palliative medicine. 2005;19(5):435-436. doi:10.1177/026921630501900515 16111072

[7] Broad JB Gott M Kim H Boyd M Chen H Connolly MJ . Where do people die? An international comparison of the percentage of deaths occurring in hospital and residential aged care settings in 45 populations, using published and available statistics. International Journal of Public Health. 2013;58(2):257-267. doi:10.1007/s00038-012-0394-5 22892713

[8] Cohen J Houttekier D Onwuteaka-Philipsen B , et al. Which patients with cancer die at home? A study of six European countries using death certificate data. J Clin Oncol: Official Journal of the American Society of Clinical Oncology. 2010;28(13):2267-2273. doi:10.1200/JCO.2009.23.2850 20351336

[9] Cohen J Bilsen J Addington-Hall J , et al. Population-based study of dying in hospital in six European countries. Palliative Medicine. 2008;22(6):702-710. doi:10.1177/0269216308092285 18715968

[10] Hakanson C Ohlen J Morin L Cohen J . A population-level study of place of death and associated factors in Sweden. Scandinavian Journal of Public Health. 2015;43(7):744-751. doi:10.1177/1403494815595774 26194351

[11] Black H Waugh C Munoz-Arroyo R , et al. Predictors of place of death in South West Scotland 2000-2010: retrospective cohort study. Palliative Medicine. 2016;30(8):764-771. doi:10.1177/0269216315627122 26857358 PMC4994701

[12] Choi KS Chae YM Lee CG , et al. Factors influencing preferences for place of terminal care and of death among cancer patients and their families in Korea. Support Care Cancer : Official Journal of the Multinational Association of Supportive Care in Cancer. 2005;13(8):565-572. doi:10.1007/s00520-005-0809-4 15812653

[13] Rasch-Westin M Helde-Frankling M Bjorkhem-Bergman L . Death at home: predictive factors in a medical home care unit. BMJ Support Palliat Care. 2019. doi:10.1136/bmjspcare-2019-001932 31537581

[14] Tang ST McCorkle R . Determinants of congruence between the preferred and actual place of death for terminally ill cancer patients. Journal of Palliative Care. 2003;19(4):230-237. 14959592

[15] Beccaro M Costantini M Giorgi Rossi P , et al. Actual and preferred place of death of cancer patients. Results from the Italian survey of the dying of cancer (ISDOC). Journal of epidemiology and community health. 2006;60(5):412-416. doi:10.1136/jech.2005.043646 16614331 PMC2563975

[16] Brogaard T Neergaard MA Sokolowski I Olesen F Jensen AB . Congruence between preferred and actual place of care and death among Danish cancer patients. *Palliative medicine*. Feb. 2013;27(2):155-164. doi:10.1177/0269216312438468 22419677

[17] Blanchard CL Ayeni O O'Neil DS , et al.. A prospective cohort study of factors associated With place of death among patients With Late-Stage cancer in Southern Africa. J Pain Symptom Manag. 2019;57(5):923-932. doi:10.1016/j.jpainsymman.2019.01.014 PMC6531674 30708125

[18] Cai J Zhang L Guerriere D Coyte PC . Congruence between preferred and actual place of death for those in receipt of home-based palliative care. Journal of Palliative Medicine. 2020;23(11):1460-1467. doi:10.1089/jpm.2019.0582 32286904

[19] Tay RY Choo RWK Ong WY Hum AYM . Predictors of the final place of care of patients with advanced cancer receiving integrated home-based palliative care: a retrospective cohort study. BMC Palliat Care. 2021;20(1):164. doi:10.1186/s12904-021-00865-5 34663303 PMC8522009

[20] Lee EJ Lee NR . Factors associated with place of death for terminal cancer patients who wished to die at home. Medicine. 2022;101(39):e30756. doi:10.1097/MD.0000000000030756 36181095 PMC9524872

[21] Valentino TCO Paiva CE de Oliveira MA , et al.. Preference and actual place-of-death in advanced cancer: Prospective longitudinal study. BMJ Support Palliat Care. 2023. doi:10.1136/spcare-2023-004299 37402541

[22] Gomes B Higginson IJ . Factors influencing death at home in terminally ill patients with cancer: Systematic review. Bmj. 2006;332(7540):515-521. doi:10.1136/bmj.38740.614954.55 16467346 PMC1388126

[23] Gomes B Calanzani N Higginson IJ . Reversal of the British trends in place of death: Time series analysis 2004-2010. Palliative Medicine. 2012;26(2):102-107. doi:10.1177/0269216311432329 22258367

