# Supplementary material for: Prospective Study of Preferred Versus Actual Place of Death Among Swedish Palliative Cancer Patients
Source: Am J Hosp Palliat Care. 2023 Nov 6;41(9):969–77. doi: 10.1177/10499091231213640 (PMC11318221; doi:10.1177/10499091231213640)
Supplement: Supplemental Material - Prospective Study of Preferred Versus Actual Place of Death Among Swedish Palliative Cancer Patients [file sj-pdf-1-ajh-10.1177_10499091231213640.pdf]

Supplemental Table 1: Questionnaire

Preferred place of death: **Date:** ___________

☐	At home
☐	Nursing home
☐	Hospital
☐	Hospice
☐	Don’t know
☐	Other: _________

Choice of preferred place of death was made:

☐	By the patient
☐	In consultation with relatives

Marital status:

☐	Married
☐	Single/Living alone
☐	Living with partner
☐	Partner in different household
☐	Other: _________

Home service:

Yes	No	
☐	☐	Domestic service
☐	☐	Home care

Need of help in relation to relatives:

☐	No need of help
☐	Take cares of self, but would like help from relatives
☐	Gets help from relatives (in the same household)
☐	Gets help from relatives (who does not live in the same household)
☐	Other: _________

Completed by:

☐	The patient him-/herself
☐	By health care staff, in conjuntion with the patient
